# Hospital Administrators at the Base Hospitals in South Africa

**Published:** 1915-02-27

**Authors:** Geo. Foster

**Affiliations:** Resident Secretary. The Government Hospital, Boksburg, Transvaal


*To the Editor of* The Hospital.

### Hospital Administrators at the Base Hospitals in South Africa.

*To the Editor of* The Hospital.

Sir,—In a recent number you made reference to the fact that in England the Government were not calling upon any of the secretary-managers of English hospitals to assist in hospital organisation work in France.

I thought I would let you know that this week I have been asked by the Department of Defence to proceed to the base hospital of 1,000 beds at Wynburg, with the rank of captain, to assist in organisation work.

I am being loaned from here for as long as my services are required, and leave to-morrow night for Wynburg, near Cape Town.—Yours faithfully,

Geo. Foster, Resident Secretary.

The Government Hospital,
Boksburg, Transvaal,
January 30, 1915.

